# The elevated risk of sight-threatening cataract in diabetes with retinopathy: a retrospective population-based cohort study

**DOI:** 10.1186/s12886-021-02114-y

**Published:** 2021-09-29

**Authors:** Chan-Wei Nien, Chia-Yi Lee, Hung-Chi Chen, Shih-Chun Chao, Hung-Jui Hsu, Shih-Hao Tzeng, Shang-Jung Yang, Jing-Yang Huang, Shun-Fa Yang, Hung-Yu Lin

**Affiliations:** 1 Institute of Medicine, Chung Shan Medical University, No. 110, Sec. 1, Chien-Kuo N. Rd., Taichung, 40201 Taiwan; 2 Department of Ophthalmology, Show Chwan Memorial Hospital, Changhua, Taiwan; 3 Department of Optometry, Central Taiwan University of Science and Technology, Taichung, Taiwan; 4 Department of Ophthalmology, Chang Gung Memorial Hospital, Linkou, Taiwan; 5 Department of Medicine, Chang Gung University College of Medicine, Taoyuan, Taiwan; 6 Center for Tissue Engineering, Chang Gung Memorial Hospital, Linkou, Taiwan; 7 Department of Electrical and Computer Engineering, National Chiao Tung University, Hsinchu, Taiwan; 8 Department of Radiology, Shuang-Ho Hospital, Taipei Medical University and School of Medicine, Zhonghe, Taiwan; 9 College of Medicine, Taipei Medical University, Taipei, Taiwan; 10 Department of Medical Research, Chung Shan Medical University Hospital, Taichung, Taiwan; 11 Department of Optometry, Chung Shan Medical University, Taichung, Taiwan; 12 College of Medicine, National Chung Hsing University, Taichung, Taiwan

**Keywords:** Diabetes mellitus, Diabetic retinopathy, Cataract surgery, Epidemiology

## Abstract

**Background:**

The effect of diabetic retinopathy (DR) on the development of sight-threatening cataracts was assessed using the National Health Insurance Research Database of Taiwan.

**Methods:**

Patients diagnosed with diabetes mellitus (DM) and DR were enrolled in the study group. Age- and sex-matched DM individuals without DR and patients without DM served as the DM control group and non-DM control group, respectively, both with 1:4 ratios. The outcome was set as the performance of cataract surgery. Cox proportional hazard regression was used to calculate the adjusted hazard ratio (aHR) of DR considering multiple factors underlying cataract formation.

**Results:**

A total of 3297 DR patients, 13,188 DM control patients and 13,188 non-DM control subjects were enrolled. The study group included 919 events of sight-threatening cataracts (27.87%), the DM control group included 1108 events (8.40%), and the non-DM control group included 957 events (7.26%). A multivariable analysis indicated that the study group presented a higher aHR of cataract surgery (2.93, 95% CI: 2.60–3.30) and a higher cumulative probability of cataract surgery than both the DM control and non-DM control groups (both log rank *P* < 0.001). In addition, both the proliferative DR (3.90, 95% CI: 3.42–4.45) and nonproliferative DR (2.35, 95% CI: 2.08–2.65) subgroups showed a higher aHR of cataract surgery than the DM control group.

**Conclusion:**

The presence of DR increases the risk of sight-threatening cataracts that warrant surgery, and the effect is prominent among patients with both proliferative DR and nonproliferative DR.

## Background

Cataracts are the leading cause of reversible blindness throughout the world [1, 2]. In a recent study conducted in Asia and Oceania, the prevalence of cataract-induced blindness and moderate-to-severe visual impairment in the general population was more than 35% [3]. The associated ocular morbidities of cataracts include glaucoma and uveitis, and they can both lead to impaired vision [4, 5]. Surgical management is always indicated for sight-threatening cataracts, although some complications may occur after surgery, such as postoperative endophthalmitis and cystoid macular oedema [6, 7].

Regarding the possible risk factors for the formation of cataracts, ultraviolet exposure has shown a significant association with cataract development [8]. In addition, trans pars plana vitrectomy may alter the oxygen concentration in the vitreous cavity and lead to subsequent cataracts [9, 10]. Both systemic and local steroid administration cause cataracts [11, 12], while certain medications, including phenothiazines, amiodarone, statins and metformin, may also increase the possibility of cataract formation [13–16]. Diabetes mellitus (DM) is a metabolic disorder featuring hyperglycaemia and insulin dysregulation, which can influence retinal and cataract development [17–21]. The risk of cataract formation can be up to fivefold in patients with DM compared to non-DM individuals [22].

Although the relationship between DM and cataract occurrence has been well established, certain issues remain to be addressed. For example, conflicting results have been obtained regarding whether the duration of DM is a risk factor for developing cataracts [18, 23]. In addition, although the presence of diabetic retinopathy (DR), which is an indicator of worse ocular condition in DM patients [19, 24], may influence the incidence of cataracts, this association has rarely been reported. Moreover, DR can be categorised into nonproliferative diabetic retinopathy (NPDR) and more severe proliferative diabetic retinopathy (PDR) [25], while the effect of different DR severities on the formation of cataracts that require surgery is also unknown.

Herein, the National Health Insurance Research Database (NHIRD) of Taiwan was used to evaluate the effect of DR, including both NPDR and PDR, on the development of cataracts for which surgical intervention was indicated to improve vision. In addition, the duration of DM and other possible potential risk factors for sight-threatening cataracts will also be surveyed in the multivariate model.

## Material and method

### Data source

The retrospective population-based cohort study was conducted in accordance with the Declaration of Helsinki and was approved by both the National Health Insurance Administration of Taiwan and the Institutional Review Board of Chung Shan Medical University (identification code: CS-17075). Supported by the Taiwan National Health Research Institutes, the NHIRD contains medical information on insurance claims from nearly the entire population of Taiwan. These claims data were obtained from the Longitudinal Health Insurance Database 2005 version (LHID), which includes information on 2 million individuals randomly selected from the NHIRD documents for 2005. The LHIDs were connected from 1 January 2000 until 31 December 2016, and the International Classification of Diseases, Ninth Revision (ICD-9) and International Classification of Diseases, Tenth Revision (ICD-10) were applied for disease diagnosis and identification. Information on the patients’ medication history, demographic characteristics, socioeconomic conditions, and locations of residence are also accessible from the NHIRD.

### Patient selection

All the subjects included in the current study were selected in 2005, and the relevant data for the participants were traced from 2000 to 2016. Patients were regarded as a case with DM if (1) their medical documents demonstrated a history of type 2 DM; (2) a blood glucose test, a glycosylated haemoglobin test, or an oral glucose tolerance test was scheduled before the diagnosis of DM; or (3) DM was diagnosed by an internal medicine or family medicine department. To more accurately elucidate the association between DM and related retinopathy and cataracts, the following criteria were applied to exclude certain impaired ocular conditions: (1) diagnosed with legal blindness before the diagnosis of DM; (2) scheduled any type of eyeball removal surgery before the diagnosis of DM; (3) receipt of a diagnosis of ophthalmic tumours before the diagnosis of DM; (4) receipt of a diagnosis of prominent ocular trauma at any time; (5) scheduled cataract surgery, diagnosed with pseudophakia, and scheduled Nd:YAG capsulotomy before the index date; (6) diagnosed with myopia, which has previously been associated with cataract formation [26]; (7) diagnosed with DR before the diagnosis of DM; (8) event is before the index date; and (9) receipt of a diagnosis of DM before 2005 (to exclude patients with extremely long DM disease periods). After exclusion, the study group was set as DR individuals who (1) had a diagnosis of DR, (2) scheduled fundus photography before the DR diagnosis and (3) scheduled optical coherence tomography before the DR diagnosis. In addition, every subject in the study group was age- and sex-matched with four DM patients without DR and non-DM individuals, which served as the DM control group and non-DM control group, respectively. Patients with DR who could not be matched with either four DM patients without DR or four non-DM patients were excluded, and the same exclusion criteria applied to the study group were also used before matching in both the DM control and non-DM control groups. The index date was regarded as the date of DR diagnosis in the study group, and the same day was also set as the index date in the matched DM control group and non-DM control group. Furthermore, the patients in the DR population were regarded as PDR if (1) NPDR-related diagnostic codes were present and (2) PDR-related codes were present with an additional diagnosis of retinal neovascularisation, vitreous haemorrhage, tractional retinal detachment or neovascular glaucoma. The parameters between the NPDR and PDR subgroups were evaluated in the following analyses.

### Main outcome measurement

The development of sight-threatening cataracts was regarded as the primary outcome in the current study, and it was based on the emergence of senile/complicated/diabetic cataract-related diagnostic codes plus the receipt of cataract surgery after the index date. Cataract-related diagnostic codes that clearly indicate the underlying aetiology were not included in the current study to prevent confusion or overestimation. In addition, only those subjects who received those diagnostic codes by an ophthalmologist were recognised as having achieved the outcome and were included in the study.

### Demographic data and comorbidities

To increase the homogeneity of the health status of each subject, we also assessed the influence of age, sex and the following systemic diseases in the multivariable analysis: hypertension, ischaemic heart diseases, hyperlipidaemia, congestive heart failure, cerebrovascular disease, dementia, chronic pulmonary disease, rheumatoid arthritis and osteoarthritis, kidney disease, liver cirrhosis, alcoholic liver disease, gout, atopic dermatitis, and allergic otolaryngologic diseases. Similarly, dry eye diseases (DEDs), uveitis, glaucoma, age-related macular degeneration (AMD) and the scheduling of trans pars plana vitrectomy (TPPV) were considered in the multivariate model to standardise the ocular condition. Since certain medications may lead to the development of cataracts, the following medications were also included in our analysis: systemic steroids, including prednisolone, methylprednisolone, hydrocortisone, triamcinolone, and dexamethasone; topical steroids, including prednisolone, fluorometholone, betamethasone, triamcinolone, dexamethasone, phenothiazines, and amiodarone; statins, including rosuvastatin, atorvastatin, simvastatin, pravastatin, lovastatin, fluvastatin and pitavastatin; and metformin. We traced the data in the NHIRD longitudinally from the index date of each participant to (1) the date of sight-threatening cataract development, (2) withdrawal from the National Health Insurance program, or (3) the time of 31 December 2016.

### Statistical analysis

SAS version 9.4 (SAS Institute Inc., NC, USA) was applied for all statistical analyses. After performing age- and sex-matching (1:4 ratio) for the study group, DM-control group and non-DM control groups, the chi-square test and independent t test were applied to compare the differences in age, sex, comorbidities and medications, including hypoglycaemic agents, between the study and control groups, and the independent t test was also used to analyse differences in ophthalmic characteristics, including the percentage of diabetic macular oedema (DME), ratio of laser photocoagulation and ratio of intravitreal injection, between the patients with NPDR and the individuals with PDR. Then, a Poisson regression was conducted to calculate the incidence rate, crude relative risk and related 95% confidence intervals (CIs). We conducted a Cox proportional hazard regression to calculate the adjusted hazard ratios (aHRs) of sight-threatening cataracts among the three groups by incorporating the aforementioned parameters in the multivariate analysis, which included age; sex; the duration of DM; the presence of hypertension, ischaemic heart diseases, hyperlipidaemia, congestive heart failure, cerebrovascular disease, dementia, chronic pulmonary disease, rheumatoid arthritis and osteoarthritis, kidney disease, liver cirrhosis, alcoholic liver disease, gout, atopic dermatitis, allergic otolaryngologic diseases, DED, uveitis, glaucoma, and AMD; scheduling of TPPV; and the use of systemic steroids (including prednisolone, methylprednisolone, hydrocortisone, triamcinolone, and dexamethasone), topical steroids (including prednisolone, fluorometholone, betamethasone, triamcinolone, dexamethasone, phenothiazines, and amiodarone), statins (including rosuvastatin, atorvastatin, simvastatin, pravastatin, lovastatin, fluvastatin and pitavastatin), and metformin. To investigate the effect of DR severity on the development of sight-threatening cataracts, the study group was further divided into NPDR and PDR subgroups in the multivariate model, and the aHR of each subgroup was analysed. Moreover, a sensitivity analysis with the aHR of sight-threatening cataracts stratified by age, sex, severity of DR and DM interval (less than 2 years, 2 to 5 years and more than 5 years) was performed for the subgroups. Additionally, we drew Kaplan–Meier curves to present the cumulative probability of sight-threatening cataracts among the study, DM control and non-DM control groups. Subsequently, the log rank test was used to evaluate the significance among all three survival curves. Since almost all the individuals enrolled in the NHIRD belong to the Han/Taiwanese population, ethnicity was thus not considered a possible confounding factor. Statistical significance was set at *P* < 0.05.

## Results

**Fig. 1:**
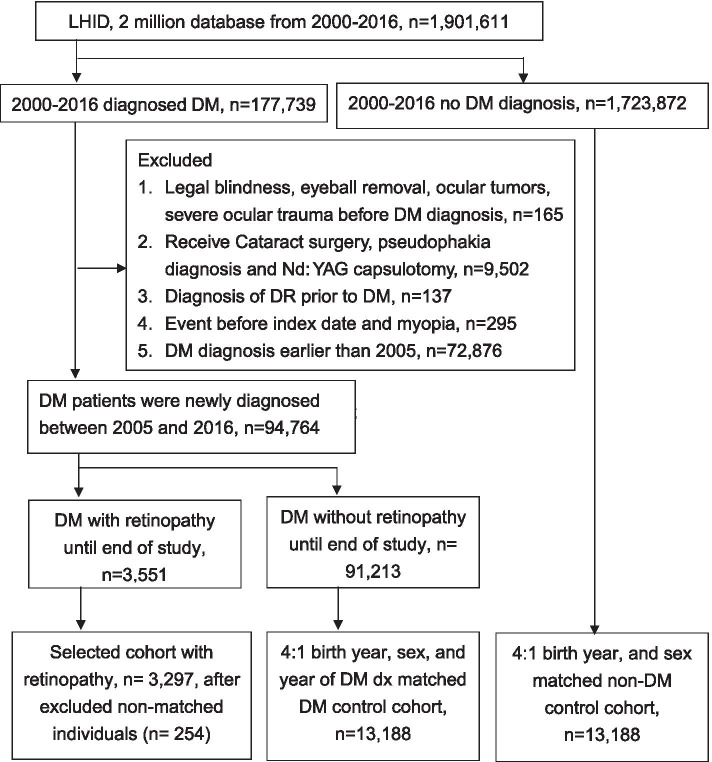
The flowchart of patients selection. LHID: Longitudinal Health Insurance Database 2005 version. DM: diabetes mellitus. DR: diabetic retinopathy

A total of 3297 DR patients, 13,188 DM control patients and 13,188 non-DM control subjects were selected for enrolment in the current study, as shown in Fig. 1. Due to the matching process, the age and gender distributions were identical among the three groups. For the potential risk factors, a significant difference in the ratio of comorbidities and medications was found among the groups except for history of dementia, chronic pulmonary disease, rheumatoid arthritis, osteoarthritis and atopic dermatitis (Table 1). Moreover, the use of oral hypoglycaemic agents and insulin was highest in the DR group (all *P* < 0.05) (Table 2) and the rates of DME, laser photocoagulation and intravitreal injection were significantly higher in the PDR population than the NPDR population (all *P* < 0.05) (Table 2).

**Table 1:** Basic characteristics among the study and control groups

Characteristics	Non-DM Control *n* = 13,188	DM Control *n* = 13,188	DR *n* = 3297	P1	P2
Age				1.0000	1.0000
< 40	672 (5.1%)	672 (5.1%)	168 (5.1%)		
40–59	7168 (54.35%)	7168 (54.35%)	1792 (54.35%)		
60–79	5048 (38.28%)	5048 (38.28%)	1262 (38.28%)		
> =80	300 (2.27%)	300 (2.27%)	75 (2.27%)		
Gender				1.0000	1.0000
Male	6872 (52.11%)	6872 (52.11%)	1718 (52.11%)		
Female	6316 (47.89%)	6316 (47.89%)	1579 (47.89%)		
Co-morbidities					
Hypertension	4441 (33.67%)	8696 (65.94%)	2321 (70.4%)	< 0.0001*	< 0.0001*
Ischemic heart diseases	1573 (11.93%)	2977 (22.57%)	653 (19.81%)	< 0.0001*	0.0006*
Hyperlipidemia	3425 (25.97%)	8751 (66.36%)	2134 (64.73%)	< 0.0001*	0.0771
Congestive heart failure	567 (4.3%)	1260 (9.55%)	324 (9.83%)	< 0.0001*	0.6343
Cerebrovascular disease	1255 (9.52%)	2135 (16.19%)	530 (16.08%)	< 0.0001*	0.8739
Dementia	193 (1.46%)	262 (1.99%)	35 (1.06%)	0.0772	0.0004*
Chronic pulmonary diseases	2736 (20.75%)	3763 (28.53%)	715 (21.69%)	0.2353	< 0.0001*
Rheumatoid arthritis as well as osteoarthritis	367 (2.78%)	442 (3.35%)	102 (3.09%)	0.3369	0.4586
Kidney disease	695 (5.27%)	1507 (11.43%)	493 (14.95%)	< 0.0001*	< 0.0001*
Hepatitis and liver cirrhosis	3080 (23.35%)	5511 (41.79%)	1032 (31.3%)	< 0.0001*	< 0.0001*
Alcoholic liver disease	125 (0.95%)	401 (3.04%)	82 (2.49%)	< 0.0001*	0.0919
Gout	1642 (12.45%)	3079 (23.35%)	547 (16.59%)	< 0.0001*	< 0.0001*
Atopic dermatitis	475 (3.6%)	667 (5.06%)	133 (4.03%)	0.2389	0.0144*
Allergic otolaryngologic diseases	2705 (20.51%)	3067 (23.26%)	563 (17.08%)	< 0.0001*	< 0.0001*
DED	1093 (8.29%)	1241 (9.41%)	466 (14.13%)	< 0.0001*	< 0.0001*
Uveitis	111 (0.84%)	122 (0.93%)	139 (4.22%)	< 0.0001*	< 0.0001*
Glaucoma	297 (2.25%)	376 (2.85%)	270 (8.19%)	< 0.0001*	< 0.0001*
AMD	130 (0.99%)	157 (1.19%)	172 (5.22%)	< 0.0001*	< 0.0001*
Retinal diseases warrant trans pars plana vitrectomy	19 (0.14%)	11 (0.08%)	181 (5.49%)	< 0.0001*	< 0.0001*
Co-medication					
Systemic steroid	1974 (14.97%)	2676 (20.29%)	752 (22.81%)	< 0.0001*	0.0014*
Topical steroid	1737 (13.17%)	2048 (15.53%)	1810 (54.9%)	< 0.0001*	< 0.0001*
Phenothiazines	385 (2.92%)	671 (5.09%)	236 (7.16%)	< 0.0001*	< 0.0001*
Amiodarone	54 (0.41%)	136 (1.03%)	26 (0.79%)	0.0051*	0.2065
Statin	993 (7.53%)	4069 (30.85%)	1348 (40.89%)	< 0.0001*	< 0.0001*
Metformin	397 (3.01%)	6558 (49.73%)	2510 (76.13%)	< 0.0001*	< 0.0001*
Other oral hypoglycemic agents	160 (1.21%)	7489 (56.79%)	2787 (84.52)	< 0.0001*	< 0.0001*
Insulin	3 (0.02%)	1536 (11.67%)	1736 (52.64%)	< 0.0001*	< 0.0001*
Duration of DM (years)				1.0000	1.0000
0	13,188 (100%)	0 (0%)	0 (0%)		
< 2	0 (0%)	6456 (48.95%)	1614 (48.95%)		
2–5	0 (0%)	3828 (29.03%)	957 (29.03%)		
≥ 5	0 (0%)	2904 (22.02%)	726 (22.02%)		
Type of DR				–	–
Without DR	13,188 (100%)	13,188 (100%)	0 (0%)		
NPDR	0 (0%)	0 (0%)	1788 (54.23%)		
PDR	0 (0%)	0 (0%)	1509 (45.77%)		

*P1* Comparison between non-diabetes mellitus control group and the diabetic retinopathy group, *P2* Comparison between diabetes mellitus control group and the diabetic retinopathy group, *DED* Dry eye disease, *AMD* Age-related macular degeneration, *DM* Diabetes mellitus, *DR* Diabetic retinopathy, *NPDR* Non-proliferative diabetic retinopathy, *PDR* Proliferative diabetic retinopathy

*denotes significant difference

**Table 2:** The characteristics between the non-proliferative diabetic retinopathy and proliferative diabetic retinopathy subgroups

Characteristics	NPDR *n* = 1788	PDR *n* = 1509	*P*
DME (n, %)	256 (14.3)	359 (23.8)	0.036*
Laser photocoagulation (n, %)	293 (16.4)	504 (33.4)	0.010*
Intravitreal injections (n, %)	299 (16.7)	1242 (82.3)	< 0.001*

*DME* Diabetic macular edema

*denotes significant difference between the two groups

**Fig. 2:**
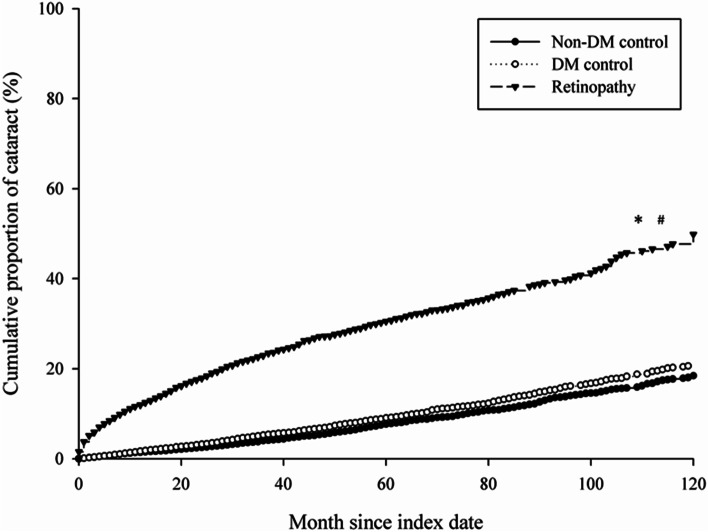
The cumulative probability of sight-threatening cataract among the study and control groups. * denotes Log rank *P* < 0.001, diabetic retinopathy versus Non-DM control. ^#^ denotes Log rank *P* < 0.001, diabetic retinopathy versus DM control

Throughout the follow-up period, 919 sight-threatening cataract events (27.87%) occurred in the study group, which presented a higher crude relative risk than the DM control group (1108 events, 8.40%) and the non-DM control group (957 events, 7.26%) (crude relative risk: 4.62, 95% CI: 4.22–5.06) (Table 3). The multivariate analysis of demographic data, systemic diseases, prominent ocular diseases and medications that may lead to sight-threatening cataracts showed that the study group presented a significantly higher aHR than the non-DM control group (2.93, 95% CI: 2.60–3.30). However, the DM control group did not reveal a higher aHR of cataract surgery performance than the non-DM control group (1.02, 95% CI: 0.92–1.12). The other risk factors for sight-threatening cataracts included age from 60 years to 79 years (aHR: 3.50, 95% CI: 3.22–3.81), age older than 80 years; a history of hypertension (aHR: 1.25, 95% CI: 1.14–1.36), ischemic heart disease (aHR: 1.12, 95% CI: 1.02–1.22), chronic pulmonary disease (aHR: 1.11, 95% CI: 1.02–1.21), rheumatoid arthritis and osteoarthritis (aHR: 1.23, 95% CI: 1.03–1.47), kidney disease (aHR: 1.22, 95% CI: 1.09–1.36), gout (aHR: 1.16, 95% CI: 1.06–1.27), atopic dermatitis (aHR: 1.19, 95% CI: 1.01–1.40), DED (aHR: 1.16, 95% CI: 1.04–1.28), uveitis (aHR: 1.34, 95% CI: 1.06–1.70), glaucoma (aHR: 1.42, 95% CI: 1.23–1.65), and AMD (aHR: 1.32, 95% CI: 1.12–1.59); retinal diseases treated with TPPV (aHR: 1.79, 95% CI: 1.48–2.24); and the use of systemic steroids (aHR: 1.16, 95% CI: 1.06–1.26) and topical steroids (aHR: 1.89, 95% CI: 1.75–2.06) (Table 4). Furthermore, the Kaplan–Meier curves revealed a significantly higher cumulative probability of sight-threatening cataracts compared to both the DM control (Log rank, *P* < 0.001) and non-DM control groups (Log rank, *P* < 0.001), while the cumulative probability between the DM control group and non-DM control groups were similar (Log rank, *P* > 0.05; Fig. 2).

**Table 3:** Incidence of sight-threatening cataract among the study and control groups

Events	Non-DM control *n* = 13,188	DM control *n* = 13,188	DR *n* = 3297
Follow up person months	680,920	656,839	142,148
New cataract case	957	1108	919
Incidence rate^a^ (95% CI)	1.41 (1.32–1.50)	1.69 (1.59–1.79)	6.47 (6.06–6.90)
Crude relative risk (95% CI)	Reference	1.20 (1.10–1.31)^b^	4.62 (4.22–5.06)^b^

*DM* Diabetes mellitus, *DR* Diabetic retinopathy, *CI* Confidential interval

^a^per 1000 person months

^b^denotes significant difference

**Table 4:** Multiple Cox proportional hazard regression for estimation of adjusted hazard ratios on sight-threatening cataract

Variables	aHR (95% CI)
Study groups (ref: Non-DM control)	
DM control	1.02 (0.92–1.12)
DR	2.93 (2.60–3.30)*
Age (years, ref.: 40–59)	
< 40	0.32 (0.22–0.47)*
60–79	3.50 (3.22–3.81)*
≥ 80	3.60 (2.91–4.43)*
Gender (ref: Female)	
Male	0.84 (0.78–0.90)*
Duration of DM (ref: < 2 years)	
2–5 years	1.12 (1.01–1.24)*
≥ 5 years	1.26 (1.10–1.44)*
Type of DR (ref: Without DR)	
NPDR	2.35 (2.08–2.65)*
PDR	3.90 (3.42–4.45)*
Co-morbidities	
Hypertension	1.25 (1.14–1.36)*
Ischemic heart diseases	1.12 (1.02–1.22)*
Hyperlipidemia	1.01 (0.93–1.10)
Congestive heart failure	1.12 (1.00–1.27)
Cerebrovascular disease	1.04 (0.94–1.14)
Dementia	0.70 (0.53–0.92)
Chronic pulmonary diseases	1.11 (1.02–1.21)*
Rheumatoid arthritis as well as osteoarthritis	1.23 (1.03–1.47)*
Kidney disease	1.22 (1.09–1.36)*
Hepatitis and liver cirrhosis	0.96 (0.88–1.04)
Alcoholic liver disease	1.00 (0.74–1.34)
Gout	1.16 (1.06–1.27)*
Atopic dermatitis	1.19 (1.01–1.40)*
Allergic otolaryngologic diseases	0.97 (0.89–1.07)
DED	1.16 (1.04–1.28)*
Uveitis	1.34 (1.06–1.70)*
Glaucoma	1.42 (1.23–1.65)*
AMD	1.32 (1.12–1.59)*
Retinal diseases warrant trans pars plana vitrectomy	1.79 (1.48–2.24)*
Co-medication	
Systemic steroid	1.16 (1.06–1.26)*
Topical steroid	1.89 (1.75–2.06)*
Phenothiazines	1.03 (0.88–1.20)
Amiodarone	1.23 (0.87–1.74)
Statin	1.03 (0.94–1.12)
Metformin	1.06 (0.97–1.16)

*aHR* Adjusted hazard ratio, *CI* Confidential interval, *DED* Dry eye disease, *AMD* Age-related macular degeneration, *DM* Diabetes mellitus, *DR* Diabetic retinopathy

*denotes significant difference

In the subgroup analysis of the DM population, the PDR subgroup showed a significantly higher aHR of cataract surgery performance (3.90, 95% CI: 3.42–4.45) than the DM control group, and the rate of cataract surgery was also higher in the NPDR subgroup than in the DM control group (aHR: 2.35, 95% CI: 2.08–2.65) (Table 4). On the other hand, the rates of sight-threatening cataracts in both the PDR and NPDR subgroups were significantly higher than those in the DM control group regardless of the DM disease intervals and sex (Table 5). Nevertheless, NPDR patients younger than 40 years old and PDR subjects older than 80 years old did not show a significantly higher aHR of sight-threatening cataracts than the DM control group (Table 5). In addition, the PDR subgroup revealed a numerically higher aHR than the NPDR subgroup if stratified by the DM duration, sex and age, except for the elderly population aged older than 80 years (Table 5).

**Table 5:** The sensitivity analysis for the adjusted hazard ratio stratified by duration of diabetes mellitus, gender and age groups

Subgroups	Incidence rate^a^ (95% CI) of Cataract			aHR1 (95% CI)	aHR2 (95% CI)
	DM control	NPDR	PDR		
**Duration of DM (Year)**					
< 2	1.47 (1.36–1.60)	3.79 (3.26–4.41)	7.76 (6.93–8.69)	2.20 (1.83–2.63)^b^	4.06 (3.39–4.88)^b^
2–5	1.89 (1.7–2.09)	5.15 (4.36–6.08)	9.63 (8.15–11.38)	2.21 (1.79–2.73)^b^	3.46 (2.71–4.41)^b^
≥ 5	2.29 (1.98–2.65)	8.04 (6.56–9.87)	13.74 (11.04–17.11)	3.07 (2.33–4.03)^b^	4.14 (2.98–5.76)^b^
P for interaction	0.3526				
**Gender subgroups**					
Male	1.35 (1.23–1.47)	4.36 (3.75–5.06)	8.30 (7.37–9.34)	2.70 (2.24–3.25)^b^	4.00 (3.29–4.86)^b^
Female	2.05 (1.9–2.21)	5.23 (4.6–5.95)	9.48 (8.36–10.75)	2.12 (1.81–2.49)^b^	3.81 (3.18–4.57)^b^
P for interaction	0.0618				
**Age at index date**					
< 40	0.22 (0.11–0.43)	0.22 (0.03–1.54)	3.96 (2.46–6.37)	1.51 (0.17–13.26)	16.99 (3.61–80.01)^b^
40–59	0.73 (0.65–0.82)	2.49 (2.07–2.99)	7.57 (6.75–8.48)	2.85 (2.26–3.58)^b^	5.96 (4.79–7.41)^b^
60–79	3.60 (3.36–3.86)	8.48 (7.53–9.57)	13.41 (11.65–15.43)	2.16 (1.86–2.51)^b^	2.72 (2.25–3.28)^b^
≥ 80	3.13 (2.21–4.43)	12.75 (8.22–19.76)	12.53 (6.74–23.29)	2.96 (1.51–5.81)^b^	2.30 (0.95–5.61)
P for interaction	< 0.0001				

*DM* Diabetes mellitus, *NPDR* Non-proliferative diabetic retinopathy, *PDR* Proliferative diabetic retinopathy, *aHR1* Non-proliferative diabetic retinopathy versus diabetes mellitus control, *aHR2* Proliferative diabetic retinopathy versus diabetes mellitus control, *aHR* Adjusted hazard ratio, *CI* Confidential interval

^a^Incidence rate, per 1000 person months

^b^denotes significant difference

## Discussion

The current study illustrates that sight-threatening cataracts are significantly correlated with patients with DR compared to those with solitary DM or without DM, and the risk is approximately threefold higher after adjusting for multiple potential risk factors. In addition, both the patients with PDR and NPDR showed a significantly higher possibility of developing sight-threatening cataracts than subjects with DM but without DR. Other possible risk factors for sight-threatening cataracts include mainly older age, cardiovascular diseases, chronic pulmonary disease, inflammatory diseases, ocular disorders and steroid use. The major pathophysiology of DM that induces cataracts is thought to be the fluid accumulation process [27]. Hyperglycaemia leads to the additional production of sorbitol from the polyol pathway for glucose metabolism in the lens [18]. Due to elevated osmotic pressure caused by the build-up of sorbitol [27], excess fluid influxes the lens, which leads to cell swelling, membrane impairment and subsequent cataracts [18]. On the other hand, DR leads to intraocular hypoxia, ischaemia and inflammation [24]. A previous review article concerning PDR indicated that reactive oxygen species, inflammatory cytokines and vascular endothelial growth factors were increased in the vitreous cavity [28], and previous laboratory studies indicated that oxidative stress is also a risk factor for cataract formation [29–31]. Therefore, DR likely influences the microenvironment in the eye more than solitary DM and results in cataract development, which is supported by the results of the current study.

Several studies have evaluated the association of DM and cataracts and unanimously indicated that DM patients present a significant risk of cataracts. In a previous review article written by Drinkwater et al., age and blood glucose levels were correlated with a higher risk of cataract formation [23]. However, another study revealed that the duration of DM is the major risk factor for the occurrence of cataracts [18]. In the current study, the study group showed a significantly higher cumulative probability (both log-rank *P* < 0.001) than the DM control group and non-DM control group after adjusting for multiple potential risk factors for cataract formation, including patient age and DM duration. To our knowledge, this is a preliminary study to illustrate that patients with DR have a significantly elevated risk of sight-threatening cataracts compared to those with DM but without ocular manifestations. Regarding the time sequence in the current study, only cataract formation after the index date was used to indicate outcome achievement; thus, a causal association seems likely. Moreover, individuals with a history of pre-existing cataracts, previous cataract surgery or Nd:YAG laser capsulotomy to retard posterior capsular opacification after cataract surgery were excluded from the current study, and we only accounted for those with senile/complicated/diabetic cataracts that needed surgery. Using these criteria to determine the effect of DR on the occurrence of sight-threatening cataracts was accurate relative to the approaches of previous studies that enrolled patients with baseline cataracts [32]. Regarding the follow-up period, the mean disease interval of DM in the current study was approximately 10 years, which was above the average compared to previous studies [23]. Thus, the significant correlation between DR and sight-threatening cataracts in the current study may include less bias.

In the multivariate analysis of the PDR, NPDR and DM control groups, individuals with PDR and NPDR revealed a significantly higher aHR of developing sight-threatening cataracts compared to the DM control group, while the aHR in the PDR subgroup was also numerically higher than that in the NPDR subgroup. Since PDR has a higher level of oxidative stress than NPDR [28], the occurrence of sight-threatening cataracts in the PDR subgroup is likely numerically higher than that in the NPDR subgroup because of the higher level of risk factors. Nevertheless, the aHR of sight-threatening cataracts was significantly elevated in the NPDR group compared to the control group, which implied that the existence of DR is a major risk factor for cataract development, whether in a nonproliferative or proliferative form, after considering multiple potential risk factors. For the subgroup analysis stratified by age, sex and DM duration, the PDR still presented the highest aHR among nearly all subgroups, which further highlighted the influence of PDR on sight-threatening cataracts. Interestingly, the aHR of PDR was much higher than that of the NPDR and DM control subgroups in the population younger than 40 years old. A possible explanation is that young patients with PDR usually experience more severe DR and a higher rate of blindness, as demonstrated in a previous study [33].

Epidemiologically, DM influences approximately 5.7% of the global population [34], while the prevalence increases to 10.3% in one Asian population [35]. Among those individuals with DM, the gross prevalence of DR is approximately 24.7% in a similar population [36]. Similarly, cataracts are the major aetiology of legal blindness in the world and account for approximately 30–50% of severe visual impairment [3]. The occurrence rate of sight-threatening cataracts in the study group was approximately 27.9%, which was threefold higher than the DM control group (8.4%) and non-DM control group (7.3%). In a previous population-based study conducted in the same region, the prevalence of self-reported cataracts was approximately 11.8% in 2013 [37], which was much lower than that in the current study. Since both DM and cataracts affect a majority of the population and DR will increase the risk of sight-threatening cataracts in the general population, strict blood sugar control and periodic ophthalmic examination in patients with DM are advocated.

Regarding the risk factors for developing sight-threatening cataracts other than DR, all the ophthalmic diseases enrolled in the multivariate analysis were correlated with the occurrence of sight-threatening cataracts. Glaucoma, uveitis and TPPV are known risk factors for cataract formation [4, 5, 9], and advanced age and inflammatory reactions in both DED and AMD may result in increased cataract development [38, 39]. Cardiovascular diseases and inflammatory diseases are the two groups of systemic diseases associated with a higher cataract occurrence, but the exact pathophysiology needs further investigation. Both systemic steroids and topical steroids are correlated with the existence of sight-threatening cataracts, which has also been proven in previous studies [11, 12]. Age is also a well-established risk factor for cataract formation [40], and the elevated aHR in individuals with older age in all the groups of the current study further supports this concept.

Certain limitations were observed in the current study. First, the retrospective study design and the use of claimed data will restrict the accuracy and standardisation of the current study. Second, the degree of ultraviolet exposure cannot be assessed by the NHIRD and thus may lead to a certain bias. In addition, blood glucose levels, as indicated by glycosylated haemoglobin test results, are not included in the NHIRD; thus, the severity of DM is only based on a history of several complications of severe DM in the multivariate analysis, such as ischaemic heart disease, cerebrovascular disease and kidney disease. Similarly, the results of fundus photography and optical coherence tomography cannot be accessed, which may decrease the accuracy of the DR diagnosis in the current study. In addition, because the demand for cataract surgery is relatively subjective, the severity of cataracts would not be consistency with that in the study population. However, photopsia caused by posterior subcapsular cataracts and irregular lenticular astigmatism could impair vision despite good visual acuity. Accordingly, all patients who need cataract surgery may experience visual impairment to some degree that affects their daily activity.

In conclusion, the presence of DR is a significant risk factor for the development of sight-threatening cataracts that require surgery to retain visual acuity after adjusting for multiple potential risk factors. Furthermore, individuals with both PDR and NPDR are at a higher risk of sight-threatening cataracts than patients with DM but without DR. On the other hand, the rate of sight-threatening cataracts is similar between patients with DM but without DR and individuals without DM. Further large-scale prospective studies to survey the effect of treating DR on the development of sight-threatening cataracts are needed.

## Data Availability

The data that support the findings of this study are available from the National Health Insurance Administration of Taiwan but restrictions apply to the availability of these data, which were used under license for the current study, and so are not publicly available. Data are however available from the authors upon reasonable request and with permission of the National Health Insurance Administration of Taiwan.

## Abbreviations

DM: Diabetes mellitus
DR: Diabetic retinopathy
NPDR: Non-proliferative diabetic retinopathy
PDR: Proliferative diabetic retinopathy
NHIRD: National Health Insurance Research Database
LHID: Longitudinal Health Insurance Database 2005 version
ICD-9: International Classification of Diseases, Ninth Revision
ICD-10: International Classification of Diseases, Tenth Revision
DED: Dry eye diseases
AMD: Age-related macular degeneration
TPPV: Trans pars plana vitrectomy
DME: Diabetic macular edema
CI: Confidence intervals
aHR: Adjusted hazard ratios
